# Cholera and ABO Blood Group: Understanding an Ancient Association

**DOI:** 10.4269/ajtmh.16-0440

**Published:** 2016-08-03

**Authors:** Jason B. Harris, Regina C. LaRocque

**Affiliations:** ^1^ Division of Infectious Diseases, Massachusetts General Hospital, Boston, Massachusetts; ^2^ Department of Pediatrics, Harvard Medical School, Boston, Massachusetts; ^3^ Department of Medicine, Harvard Medical School, Boston, Massachusetts

Since the association was first recognized by Barua and Paguio in 1977,^1^ many investigators have confirmed a strong link between cholera and the human blood group O phenotype. Although the blood type O does not affect the risk of being infected with *Vibrio cholerae*, it has a tremendous impact on disease severity.^2^ For example, when cholera was introduced into Peru in 1991, individuals with blood type O were eight times more likely to be hospitalized with severe cholera.^3^ Thus, for individuals infected with *V. cholerae*, blood type can be a life or death matter.

It is also very likely that the association between severe cholera and blood type O has impacted our evolutionary history. The lowest prevalence of the O blood group phenotype in the world is in the Ganges delta, where cholera has likely been endemic for centuries.^4^ This suggests that cholera has left a genetic imprint in historically endemic areas, and means that as cholera spreads to areas with a higher prevalence of the blood type O, the proportion of individuals with severe forms of cholera will be higher.

In this issue of the *American Journal of Tropical Medicine and Hygiene*, F. Matthew Kuhlmann and others at Washington University take a step forward toward understanding the mechanism of the association between blood group and cholera severity.^5^ The authors studied the effect of cholera toxin on enteroids—a cell culture model derived from ileal and colonic stem cells that differentiate in vitro into tissue that mimics many features of the intestinal surface. Using this approach, the team compared the responses of cholera toxin-stimulated enteroids derived from blood type O donors to responses of identically stimulated enteroids from blood group A donors. The investigators demonstrated a significantly greater cyclic adenosine monophosphate response to cholera toxin in enteroids derived from blood type O stem cells. The team then demonstrated a similar effect after converting a blood type A-derived cell line to a blood type O phenotypic cell, using clustered regularly interspaced short palindromic repeats technology to alter the gene that controls glycosylation of the ABO blood group glycans.

Taken together, these results provide very convincing evidence that cholera toxin exerts a more potent effect on cells expressing the blood type O-associated glycan. This fits with the epidemiologic observation that blood type O only impacts disease severity, but not the risk of infection. However, this work still does not identify the precise mechanism by which cholera toxin induces a stronger response in the type O cells. In fact, a surprising finding in this study was that the greater effect of cholera toxin in blood group O-derived cells did not appear to be linked to the amount of toxin binding to cells, which was similar in both the type A- and type O-derived cells.

The Kuhlmann study coincides with interesting work recently published by Heggelund and others, who reported the first high-resolution crystal structure of cholera toxin bound to the A and O blood group glycans.^6^ Although Heggelund and others did not evaluate the cellular response to cholera toxin, the authors demonstrated that the toxin binds to the blood group O determinant in multiple orientations and with greater affinity than it binds to the blood group A determinants.

What makes this story even more intriguing is that the ABO blood group determinant is not the primary receptor for cholera toxin. Instead, the cellular response to cholera toxin is derived from the high-affinity binding of the toxin to the cognate GM1 ganglioside receptor. Thus, it is not clear how the affinity and orientation of binding to the ABO-related blood antigens impacts the cellular response to cholera toxin. Perhaps a final step in tying this together at a mechanistic level will be understanding how variations in the ABO blood group glycans impact the interaction between cholera toxin and the GM1 ganglioside. For example, this could be dependent on toxin binding, but could also be dependent on specific cell surface interactions between GM1 ganglioside and the ABO blood group glycans. Thus, it seems likely that there is still more to learn. Hopefully, an improved understanding of the molecular mechanisms of this long-observed genetic predisposition to this ancient disease will lead to new approaches for combating this globally important pathogen.
